# First person – Eleftheria Pervolaraki

**DOI:** 10.1242/dmm.042408

**Published:** 2019-09-01

## Abstract

First Person is a series of interviews with the first authors of a selection of papers published in Disease Models & Mechanisms (DMM), helping early-career researchers promote themselves alongside their papers. Eleftheria Pervolaraki is first author on ‘Insoluble Aβ overexpression in an *App* knock-in mouse model alters microstructure and gamma oscillations in the prefrontal cortex, affecting anxiety-related behaviours’, published in DMM. Eleftheria conducted the research described in this article while a Research Fellow at the School of Biomedical Sciences, University of Leeds, UK. She is now Medical Science Lead for the UK at Cardinal Health, High Wycombe, Buckinghamshire, UK, working on medical devices for the management of cardiac rhythm whilst preventing infections in surgical sites.


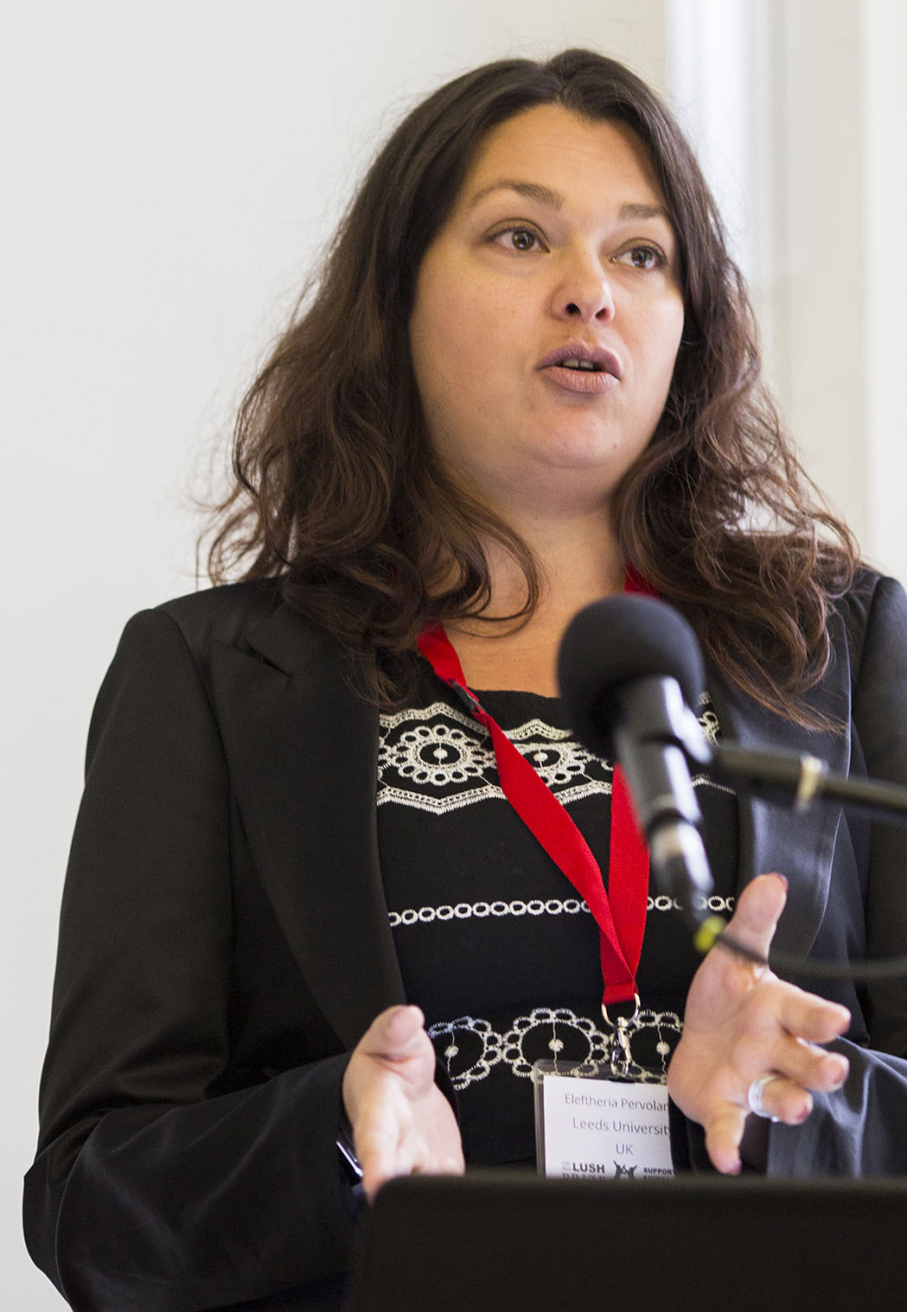


**Eleftheria Pervolaraki**

**How would you explain the main findings of your paper to non-scientific family and friends?**

Alzheimer's disease (AD) is classically associated with memory impairments. However, AD causes several other issues such as anxiety and social withdrawal. Anxiety is the second most common change to behaviour in AD patients, with up to 70% suffering some form of anxiety trait. Social withdrawal is a rarer but equally debilitating behavioural change in AD. Clearly, these behavioural changes only further compound the emotional toll that AD has on a patient and their family. Despite this, comparatively little research is undertaken into how AD impacts upon anxiety and social behaviour.

Normally, we know that anxiety and social behaviour are supported by several of the same brain regions. These include regions called the amygdala, the prefrontal cortex and the hippocampus. We also know that these particular brain regions are susceptible to AD damage, namely amyloid beta (Aβ) plaques. Furthermore, Aβ plaques are evident in these brain regions in early AD, suggesting that these regions degenerate early in the disease.

Therefore, we decided to explore whether anxiety and social behavioural impairments exist in the latest generation of AD mouse model, called the *App^NL-G-F^* mouse. This new generation of AD mouse model has substantial advantages over previous, early generation, mouse models, as their design produces damage that is more like human AD.

We performed behavioural tests on *App^NL-G-F^* mice. We found that in one anxiety test, *App^NL-G-F^* mice avoided open areas and instead preferred to spend their time next to the walls. This is indicative of anxiety behaviour. We tested the motivation for *App^NL-G-F^* mice to explore a new mouse that they have never seen before. *App^NL-G-F^* mice spent the same amount of time exploring a novel mouse as control mice, suggesting they do not lack social motivation. We then tested their ability to remember a previously explored mouse compared to a new mouse. Again, *App^NL-G-F^* mice were able to distinguish between novel and previously explored mice. However, female *App^NL-G-F^* mice were less motivated to explore a social smell, suggesting a subtle social deficit. In summary, we found that *App^NL-G-F^* mice had increased anxiety in certain circumstances and a mild social impairment, which is similar to that of AD.

To further explore why these behavioural issues occurred, we focused on the amygdala and prefrontal cortex. Using a special form of MRI called diffusion tensor MRI (DTI) that is able to examine the organization of brain tissue, we found that *App^NL-G-F^* mice only had altered neural tissue in the prefrontal cortex. The amygdala was unaffected. Next, we recorded groups of neurons to see whether brain rhythms (important for social behaviour and anxiety) were impaired in *App^NL-G-F^* mice. Again, we found that only brain rhythms in the prefrontal cortex were altered. Finally, we tried to find out what damage Aβ was doing to nerve cells within the prefrontal cortex. To do this, we measured gene expression for parts of the neuron that we knew supported normal brain rhythms. We found that expression of a gene called *Grin2b* was reduced. Interestingly, this gene has been shown to be important for both social and anxiety behaviour.

In conclusion, our study has revealed that AD pathology may cause both social and anxiety impairments, and these problems could be linked to damage to the prefrontal cortex.

**Figure:**
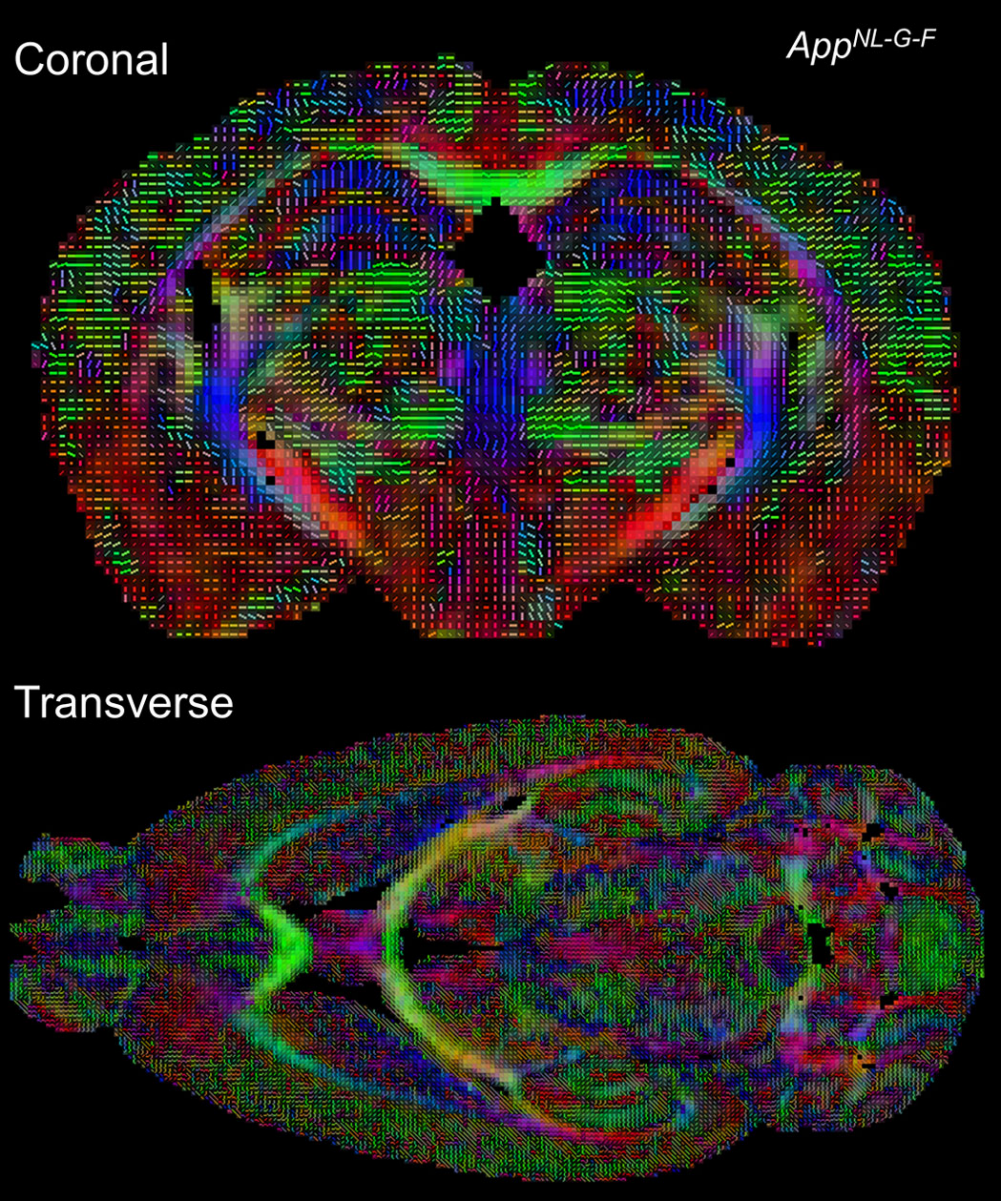
**Tractography of the *App^NL-G-F^* knock-in mouse model brain.**

**What are the potential implications of these results for your field of research?**

AD/dementia is now the leading cause of death in several countries, including the UK. Increasingly, research is starting to take into account behavioural impairments that are not solely focused upon cognitive impairment. Thus, I see three potential implications of the current study.

First, this study will increase the interest in non-cognitive AD research. Very few animal model studies have focused upon anxiety and social deficits, and our work, along with other studies using the *App^NL-G-F^* mouse, will hopefully demonstrate that this is a useful model to further explore anxiety behaviour in AD.

Second, we find a complex anxiety and social phenotype in the *App^NL-G-F^* mouse. Therefore, we hope that further studies will use a battery of tests to explore both social and anxiety behaviour, to gain a richer dataset that will enable informed conclusions.

Third, our study is one of the first to provide a rich, multidisciplinary exploration of the *App^NL-G-F^* mouse encompassing behaviour, electrophysiology, DTI and genetics. We hope that our findings, particularly those focusing on the prefrontal cortex, will enable other groups to facilitate their own research aims.

“We hope that our findings […] will enable other groups to facilitate their own research aims.”

**What are the main advantages and drawbacks of the model system you have used as it relates to the disease you are investigating?**

The problems with first-generation Aβ precursor protein (APP) transgenic mice have been well established. In short, many AD mouse models achieve elevated Aβ by overexpressing APP, and then relying upon familial AD mutations to overexpress Aβ. In AD, APP expression is similar between AD and control subjects, with mutations only elevating Aβ_40_ and Aβ_42_. The effects of APP overexpression have been studied in mice, and the phenotype is strikingly similar to what one would expect to find as a result of AD pathology, both in terms of behaviour and molecular biology of excitatory synapses. Hence, dissociating the effect of Aβ from APP overexpression has been challenging, especially when many researchers use wild-type and not APP-overexpressing mice as controls.

The new generation of mice called *App^NL-G-F^* created by Saito and colleagues (co-authors) express APP at endogenous levels, with only Aβ being elevated. This makes interpretations about the effects of Aβ more relevant to AD.

Despite this, there are still limitations. These mice only examine the effects of Aβ, meaning that the effects of tau are not represented. Second, the *App^NL-G-F^* mice are generated by three familial mutations (Swedish KM670/671NL, the Iberian I716F and the Artic E693G), which will all act slightly differently upon Aβ (e.g. promoting oligomerization). However, three mutations are not necessarily reflective of clinical AD, and certainly does not model sporadic AD.

**What has surprised you the most while conducting your research?**

We originally predicted that most of the changes would be focused upon the amygdala. This is because, behaviourally, social and anxiety behaviours are known to be mediated by the amygdala. However, DTI, electrophysiology and gene expression revealed that the majority of our findings centred upon the prefrontal cortex. The prefrontal cortex receives relatively little attention compared to the hippocampus, so we hope that more labs will focus their energy to research AD pathology outside of the hippocampus.

**Describe what you think is the most significant challenge impacting your research at this time and how will this be addressed over the next 10 years?**

AD research is somewhat at a crossroads. Many clinical trials have failed recently. Animal models have borne some of the blame, however, cell-culture models must also accept their limitations. A current problem is that a large proportion of the model systems in AD research are based upon single aspects of the AD pathology (e.g. only focus upon Aβ). Additionally, Aβ models are mostly generated by familial mutations, which represent only a fraction of all AD cases. Over the next decade, the development of better model systems will facilitate more clinically relevant discoveries. There is hope on the horizon. 3D cell-culture systems will provide a more ‘relevant’ basis for drug discovery. The mice used in our current study are a step forward, and hopefully these will incorporate tau pathologies in the future. With better models, I hope that drug translation will improve.

**What changes do you think could improve the professional lives of early-career scientists?**

Early-career scientists need more mentoring by academics and other fellows outside of their own discipline. It would also be beneficial to see other professionals (such as company representatives or industry) organize workshops where early-career scientists can ask questions about career development and opportunities. There are a lot of different avenues for personal development but very little information is available or communicated on how to achieve them. A key issue is that universities do not have the experience of how to mentor people for a career outside of academia and are often not motivated to invest significant amounts of time in providing this. Workshops from independent mentors should be offered within universities to early-career scientists. I was very lucky to attend the Biophysics Conference in early 2019, where I participated in workshops about careers and how to pursue opportunities outside academia. I found the information very useful and used it to guide my search for new opportunities. This has facilitated me to join one of the largest healthcare companies in the world.

“Workshops from independent mentors should be offered within universities to early-career scientists.”

**What's next for you?**

I am currently the Medical Science Lead in the UK for Cardinal Health, working on medical devices for the management of cardiac rhythm whilst preventing infections in surgical sites. My aim is to raise awareness surrounding the problems of current medical practices and offer sustainable solutions. I conduct research within the NHS and work closely with consultants from interventional cardiology units to identify vectors of infection on cardiology patients and offer solutions. My aim is to be the point of contact for clinical professionals, and be able to offer solutions to current problems when caring for patients.
